# Fabrication and Characterization of Thermoresponsive Polystyrene Nanofibrous Mats for Cultured Cell Recovery

**DOI:** 10.1155/2014/480694

**Published:** 2014-02-20

**Authors:** Hwan Hee Oh, Young-Gwang Ko, Hiroshi Uyama, Won Ho Park, Donghwan Cho, Oh Hyeong Kwon

**Affiliations:** ^1^Department of Polymer Science and Engineering, Kumoh National Institute of Technology, 1 Yangho-dong, Gumi, Gyeongbuk 730-701, Republic of Korea; ^2^Department of Applied Chemistry, Graduate School of Engineering, Osaka University Suita, Osaka 565-0871, Japan; ^3^Department of Advanced Organic Materials and Textile System Engineering, Chungnam National University, 79 Daehangno, Yuseong-gu, Daejeon 305-764, Republic of Korea

## Abstract

Rapid cell growth and rapid recovery of intact cultured cells are an invaluable technique to maintain the biological functions and viability of cells. To achieve this goal, thermoresponsive polystyrene (PS) nanofibrous mat was fabricated by electrospinning of PS solution, followed by the graft polymerization of thermoresponsive poly(*N*-isopropylacrylamide)(PIPAAm) on PS nanofibrous mats. Image analysis of the PS nanofiber revealed a unimodal distribution pattern with 400 nm average fiber diameter. Graft polymerization of PIPAAm on PS nanofibrous mats was confirmed by spectroscopic methods such as ATR-FTIR, ESCA, and AFM. Human fibroblasts were cultured on four different surfaces, PIPAAm-grafted and ungrafted PS dishes and PIPAAm-grafted and ungrafted PS nanofibrous mats, respectively. Cells on PIPAAm-grafted PS nanofibrous mats were well attached, spread, and proliferated significantly much more than those on other surfaces. Cultured cells were easily detached from the PIPAAm-grafted surfaces by decreasing culture temperature to 20°C, while negligible cells were detached from ungrafted surfaces. Moreover, cells on PIPAAm-grafted PS nanofibrous mats were detached more rapidly than those on PIPAAm-grafted PS dishes. These results suggest that thermoresponsive nanofibrous mats are attractive cell culture substrates which enable rapid cell growth and recovery from the culture surface for application to tissue engineering and regenerative medicine.

## 1. Introduction

Tissue engineering is a rapidly expanding field that seeks to create specific human tissues and organs by combining cells and scaffolds formed typically using either synthetic or naturally-derived polymers [1–4]. Tissue engineering has three essential components as follows: cells, scaffolds using biomaterials, and bioactive molecules. The most important component among them is highly functional cells, because it is unattainable to develop therapeutic replacement tissue even if the ideal scaffold was prepared when cells lost their natural functionality.

In this point of view, the process of cell culture requires a method to recover cells from the culture surface. Trypsin, an enzyme commonly found in the digestive tract, can be used to digest proteins, which cleaves adhesion of cells to the surface and cell-cell junctions. Trypsinization process for cultured cell recovery has a big problem [5, 6]. Protease like trypsin dissociates cell membrane proteins and secreted ECM by cells, resulting in decreased specific cell functions and viability, especially on highly differentiated functionalized cell types. To remedy this problem and recover intact cultured cells or cell sheets, thermoresponsive poly(*N*-isopropylacrylamide) (PIPAAm)-grafted PS dishes were mainly used by Okano group [7–11]. PIPAAm exhibits a phase separation behavior in water below its lower critical solution temperature (LCST) at 32°C [12]. The PIPAAm-grafted surface can reversibly change its surface hydrophilic/hydrophobic properties in response to temperature changes. Accordingly, cells can be attached to the PIPAAm-grafted surface above 32°C and detached below 32°C by hydration of grafted PIPAAm chains. Okano and his colleagues have reported many scientific and clinical results utilizing PIPAAm-grafted PS cell culture dishes [13–15]. However, cell detachment from PIPAAm-grafted PS dish surface is relatively a slow process. Rapid recovery of cultured cells is very important to prevent their functional damage, because lower temperature treatment for a long time might have negative effects on cell functions. We have already reported that cells or cell sheets cultured on PIPAAm-grafted porous membranes can be recovered more rapidly than PIPAAm-grafted nonporous PS surfaces [16, 17]. Cells can be detached by the hydration of grafted PIPAAm chains, which are promoted by the supply of essential water molecules to hydrate PIPAAm-grafted layer. With porous membranes, the water accesses the PIPAAm-grafted surface from underneath and periphery of the attached cells, resulting in rapid hydration of PIPAAm chains and cell detachment.

Recently, electrospun nanofibers have attracted great attention as a new type of scaffolds for tissue engineering [18–24]. It is well known that extracellular environment influences many aspects of cell behavior such as morphology, functionality, and cell-cell interactions [20]. In natural tissues, cells are surrounded by extracellular matrix (ECM), which has structural features ranging from nanometer to micrometer scale. Hence, a nanostructured porous and large surface area is needed as an alternate to natural ECM. To mimic the natural ECM structure, electrospinning is thought to be one of the most suitable methods to fabricate nanofibrous matrices. We have previously reported that cells on highly porous electrospun nanofibrous mat were well attached, spread, and proliferated much more than nonporous surfaces [25–30]. It is considered that highly porous nanofibrous mat with high surface area and nanosized roughness offers a biomimicking structure during cell culture, more structural space for accommodation and attachment and proliferation of cells, and enables the efficient exchange of nutrient and metabolic wastes.

Rapid cell growth and rapid recovery of intact cultured cells are an invaluable technique to maintain the biological functions and viability of cells for tissue engineering and regenerative medicine. In the present study, we fabricated thermoresponsive PS nanofibrous mats to achieve this goal by electrospinning method and subsequent surface graft polymerization of PIPAAm by electron beam irradiation.

## 2. Experimental

### 2.1. Materials

Isopropyl acrylamide (IPAAm) was purchased from TCI Chemicals (Tokyo, Japan) and used after recrystallization from *n*-hexane. Polystyrene (PS, Mw: 1,280,000; Mw/Mn = 1.03) was purchased from Polymer Source. Inc (Montreal, Canada). N,N-Dimethylformamide (DMF) to prepare PS solution was obtained from Daejung Chemicals and Metals (Gyeonggi, Korea) and used as received without further purification. Polystyrene dish (35 × 10 mm) was purchased from SPL Life Science (Gyeonggi, Korea). Trypsin-EDTA solution, streptomycin, penicillin, and Dulbecco's modified Eagle's medium (DMEM) were bought from Gibco BRL (Grand Island, NY, USA).

### 2.2. Preparation of PS Nanofibrous Mats

Nanofibrous PS mats were fabricated by electrospinning technique as previously reported [30]. Briefly, PS was dissolved in DMF at a concentration of 3 wt%. The PS solution is contained in a glass syringe controlled by syringe pump. A high voltage is applied between syringe needle and collector. When the electric field reached a critical value with increasing voltage, mutual charge repulsion overcame the surface tension of polymer solution and an electrically charged jet was ejected from the syringe needle to the collector. Because of charge repulsion in polymer solution, diameter of fibers significantly decreased during the flight. PS nanofibrous mats were fabricated reproducibly under the voltage of 10 kV, tip-to-collector distance of 15 cm and flow rate of 1 mL/h. Electrospun PS nanofibrous mat was carefully detached from collector and dried *in vacuo* for 2 days at 30°C to remove residual solvent completely.

### 2.3. Heat Treatment of PS Nanofibrous Mat

Because electrospun PS nanofibrous mat has no bonding points between fibers, its mechanical strength was too low to handle it. To remedy this problem, heat treatment was employed. PS nanofibrous mat was placed between two glass plates (20 cm × 20 cm × 3 mm) and then kept heated for 25 minutes at heating oven fixed at 120°C, which is slightly higher temperature than glass transition temperature (Tg) of PS.

### 2.4. PIPAAm Graft Polymerization on PS Nanofibrous Mat

IPAAm monomer was dissolved in 2-propanol at a concentration of 55 wt%. This monomer solution (40 *μ*L) was spread uniformly over the surface of the PS nanofibrous mat and PS dish, and then electron beam was irradiated using an area beam electron processing system (Curetron BBC-200-AA2, Nissin-High Voltage, Kyoto, Japan) at various radiation doses (acceleration voltage of 150 kV under 1.0 × 10^−4^ Pa). Unreacted monomer and ungrafted polymers were removed by washing extensively with cold water, and the PIPAAm-grafted PS matrices were dried *in vacuo* at room temperature.

### 2.5. Characterizations

The morphology and diameter of electrospun nanofibers were determined by SEM and image analyzer. Mechanical properties of PS nanofibrous mat before and after heat treatment were tested by universal testing machine. The specimen was prepared in accordance with ASTM D638. Grafting of PIPAAm on PS nanofibrous mats and PS dishes was confirmed by attenuated total reflection-Fourier transform IR (ATR-FTIR) and electron spectroscopy for chemical analysis (ESCA). The density of PIPAAm grafted onto the PS nanofibrous mats and PS dishes was determined by ATR-FTIR in comparison with standard calibration curve. The control PS substrate has strong absorption bands attributed to aromatic groups at 1600 cm^−1^. As PIPAAm was grafted onto PS surface, an amide I absorption band appeared in the region of 1650 cm^−1^. The peak intensity ratio (*I*_1650/1600_) was used to determine the amount of PIPAAm grafted on PS surface using a calibration curve of known PIPAAm amount cast on PS surface from solution. Water contact angles were determined by a sessile drop method at 20 and 37°C. Each sample was cut in size (1.0 × 1.0 cm) to measure water contact angles. All samples were measured six times and averaged. Contact angles were presented as a mean value (*n* = 6) with a standard deviation.

### 2.6. Cell Culture

To examine the tissue compatibility, human fibroblasts were evenly seeded at 20,000 cells/dish onto each surface of PIPAAm-grafted PS nanofibrous mats, ungrafted PS nanofibrous mats, PIPAAm-grafted PS dishes, and ungrafted PS dishes. Seeded fibroblasts were cultivated in Dulbecco's modified Eagle's medium (DMEM) supplemented with 10% fetal bovine serum and 1% penicillin G-streptomycin. Attached cell morphology and viability of fibroblasts were measured by SEM and MTT assay.

### 2.7. Recovery of Cultured Cells

Detachment of single cells was achieved by lower temperature treatment after incubation at 37°C for 5 hours. For lower temperature treatment, spread cells on each surface were transferred to a CO_2_ incubator equipped with a cooling unit fixed at 20°C. The morphology and detachment rate were determined with SEM and MTT assay as a function of lower temperature treatment time.

## 3. Results and Discussion

### 3.1. Characterization of Electrospun Mat

**Figure 1:**
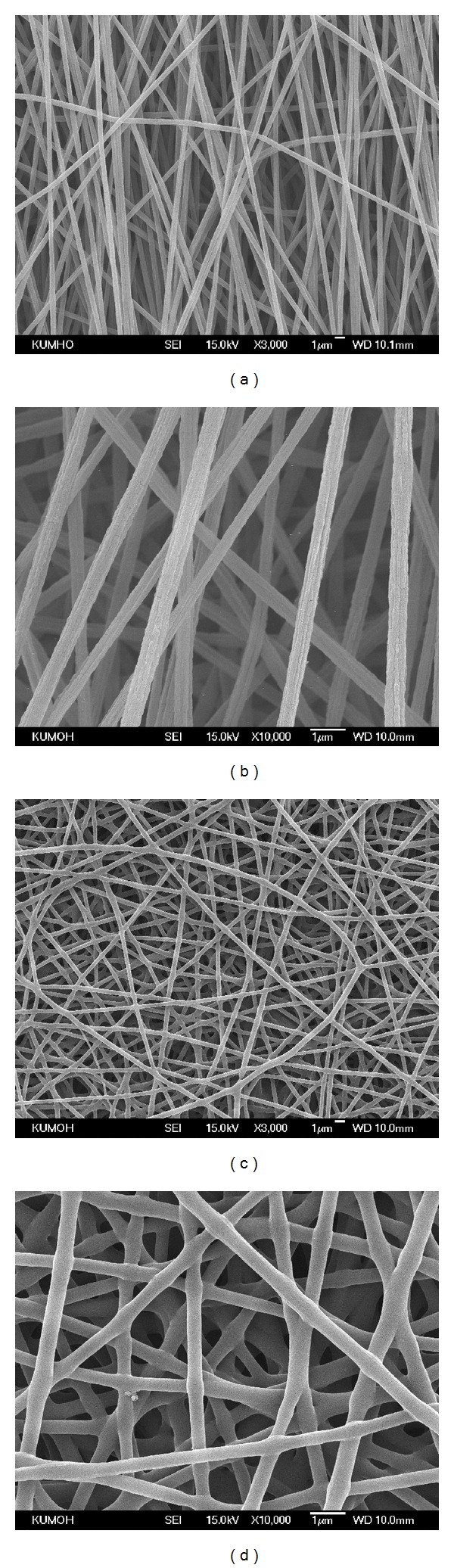
SEM micrographs of electrospun polystyrene nanofibrous mat, magnification of ×3,000 (a) and ×10,000 (b), respectively. Polystyrene nanofibrous mat after interfiber bonding treatment, magnification of ×3,000 (c) and ×10,000 (d), respectively.

Polystyrene (PS) nanofibrous mats were prepared via electrospinning with optimized conditions to have an average diameter less than 500 nm to prevent cell penetration into the mat. Electrospun PS mat structures revealed randomly aligned fibers with average diameter of 400 nm (Figures 1(a) and 1(b)). The surface of electrospun nanofibrous mats required heat treatment because of weak mechanical properties. It causes exfoliation of surface layer of nonwoven PS nanofibrous mat during washing process of poly(*N*-isopropylacrylamide) (PIPAAm)-grafted surfaces. For this reason, PS mat was heat-treated at 120°C (slightly higher temperature than glass transition temperature of the PS mat). After heat treatment, the average diameter of nanofibers was increased to 450 nm and physical crosslinking points appeared (Figures 1(c) and 1(d)). The heat-treated PS nanofibrous mat showed network structure between fibers, while the original PS nanofibrous mat showed a random straightforward structure.

**Figure 2:**
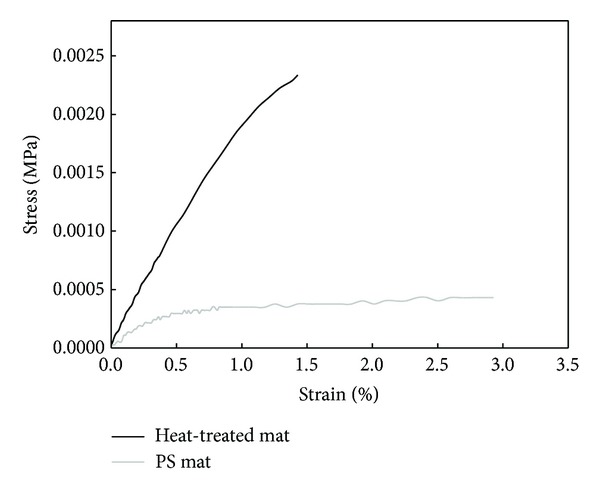
Stress-strain curves obtained from tensile test for heat-treated PS mat and original PS mat.

Mechanical properties of the heat-treated PS mat and original PS mat could be compared by using universal testing machine (UTM), and the results of tensile strength were indicated in Figure 2. Heat-treated PS mat could resist more stress than PS mat during rinsing process of PIPAAm-grafted PS mat surfaces without separation of outer surfaces. The heat-treated PS mat (148.55 ± 10.55 MPa) showed higher modulus than original PS mat (68.88 ± 7.96 MPa). We assumed that this difference of curve shape is due to a presence of physical crosslinking points on the electrospun mat. The load was transferred and absorbed into crosslinking points which appeared after heat treatment. The heat-treated PS mat was altered to be a little bit stiff and rigid than before heat treatment. However, heat treatment of PS mat facilitates washing procedure after PIPAAm graft polymerization.

### 3.2. Investigation of PIPAAm-Grafted Surfaces

**Figure 3:**
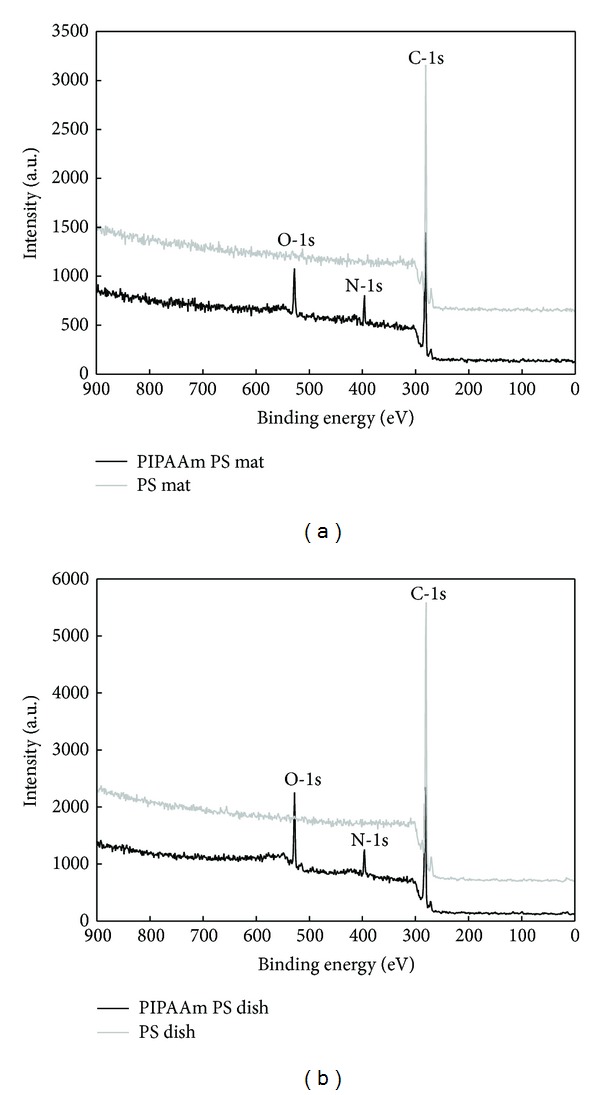
ESCA survey scan spectra of PIPAAm-grafted and ungrafted polystyrene mat (a) and PIPAAm-grafted and ungrafted polystyrene dish (b).

The PIPAAm-grafted PS mats and dishes were prepared with various conditions of electron beam irradiation. Grafting of PIPAAm onto surface was performed after preliminary examination for arranging of the irradiation dosage. There was not a morphological deformation of PS nanofibrous mats by electron beam irradiation. To confirm grafting of PIPAAm on PS surface by electron beam irradiation, surface elemental analysis was performed using ESCA. In Figure 3, an atomic percent of nitrogen was observed on the PIPAAm-grafted PS surfaces, while nitrogen was not detected on ungrafted PS surfaces. PIPAAm-grafted PS mat (Figure 3(a)) showed 79.5% of C, 8.8% of N, and 11.7% of O atomic composition. And PIPAAm-grafted PS dish (Figure 3(b)) showed 76.5% of C, 8.3% of N, and 15.2% of O atomic composition, while the ungrafted PS mat and ungrafted PS dish showed 100% of C atomic composition without N and O. Because PS does not contain amide group, nitrogen on the ungrafted surfaces was not surveyed, while PIPAAm has amide groups in the chain. From these results, we infer that PIPAAm was successfully grafted on PS mat and PS dish surfaces by electron beam irradiation.

**Figure 4:**
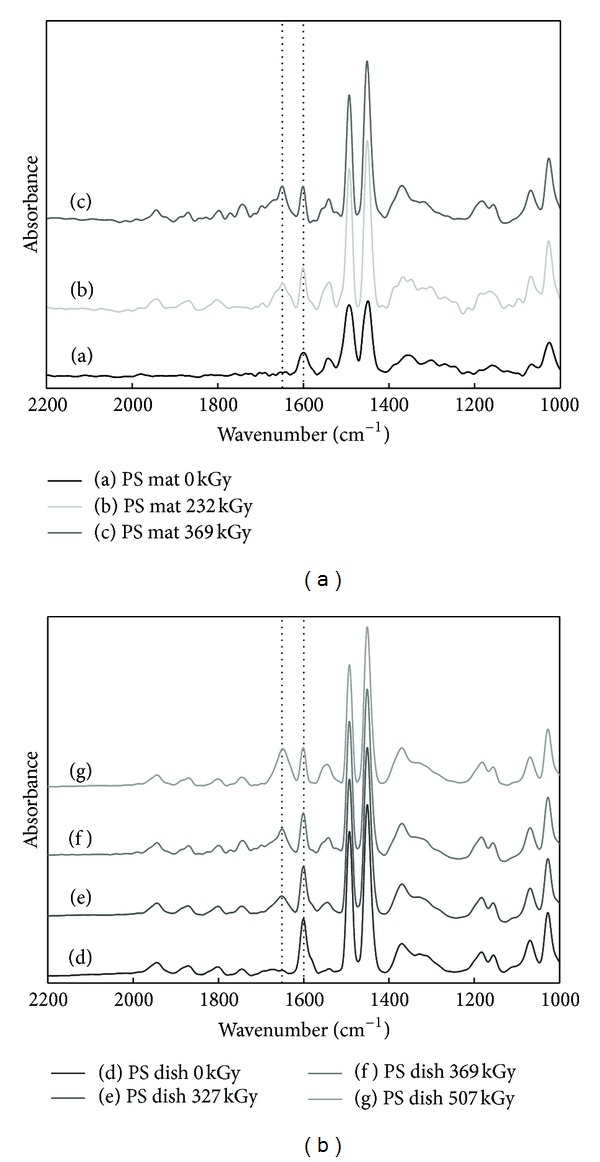
ATR-FTIR spectra of PIPAAm-grafted polystyrene mats (a) and polystyrene dishes (b) as a function of radiation dose.

Figure 4 shows ATR-FTIR spectra of PIPAAm-grafted and ungrafted PS surfaces. Significant increase of amide peak at 1650 cm^−1^ appeared at PIPAAm-grafted surfaces. PS includes aromatic groups that have a characteristic peak at 1600 cm^−1^ and a characteristic peak of PIPAAm appeared at 1650 cm^−1^ attributed to amide group in IPAAm chain. In the cases of PS mat and PS dish without electron beam irradiation (0 kGy), the absorption peak was revealed at 1600 cm^−1^ only, while PIPAAm-grafted PS mat surface by radiation dose 232, 369 kGy revealed clear peak at 1650 cm^−1^. The peak of 1650 cm^−1^ was increasing as a function of irradiation dosage (Figure 4(a)). Also in PS dishes, amide peak at 1650 cm^−1^ shows up only at PIPAAm-grafted surfaces and the peak was increased by increasing irradiation strength (Figure 4(b)). It is in accordance with ESCA results of PIPAAm-grafted PS surfaces compared with ungrafted PS surfaces. Grafted amount of PIPAAm on PS dishes and PS mats was analyzed by calculation of peak intensity at 1650 cm^−1^ and 1600 cm^−1^. The peak intensity ratio (*I*_1650_/*I*_1600_) was used to determine the graft density of PIPAAm on the surface using the calibration curve. PIPAAm-grafted PS mat with 369 kGy irradiation and PIPAAm-grafted PS dish with 507 kGy irradiation were grafted approximately 1.02 g/cm^2^ and 1.01 g/cm^2^ of PIPAAm, respectively.

**Figure 5:**
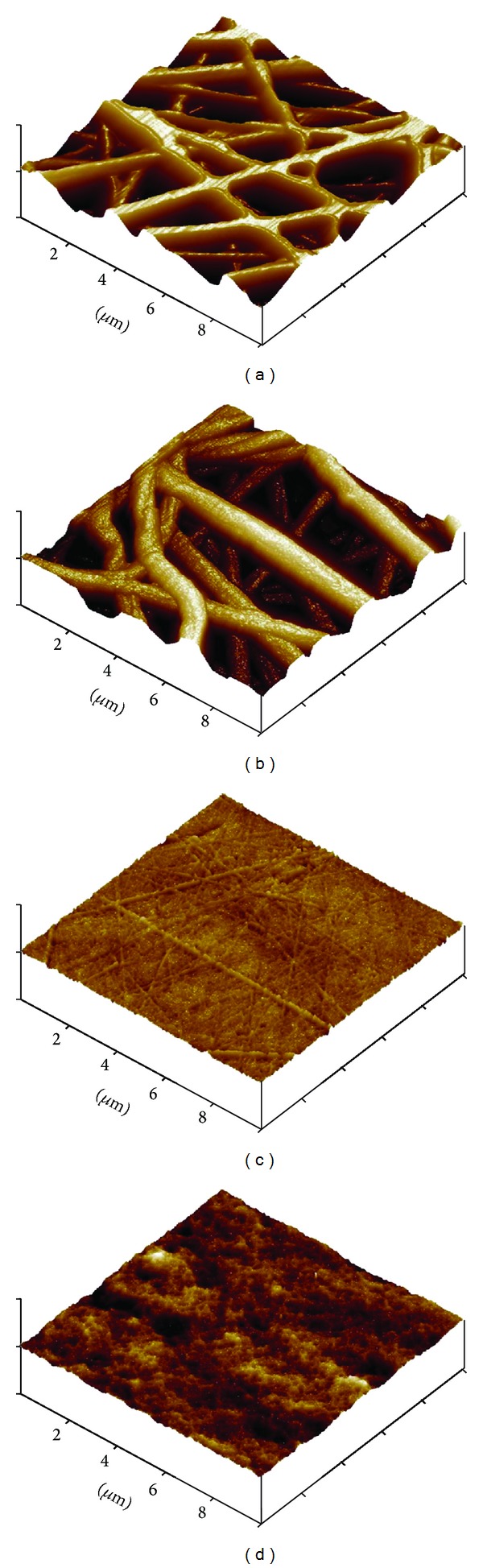
Three-dimensional tapping mode AFM topographical images of each surface: (a) ungrafted polystyrene mat, (b) PIPAAm-grafted polystyrene mat, (c) ungrafted polystyrene film, and (d) PIPAAm-grafted polystyrene dish.

The topography of polystyrene surfaces was measured by tapping mode of atomic force microscope (AFM) (Figure 5). PS nanofibrous mat showed randomly overlapped logs-like structure (Figure 5(b)) and PS dish showed a lawn-like morphology (Figure 5(d)). The morphology of PIPAAm-grafted surfaces was altered to rough surface compared to neat PS surfaces. In the case of nanofibrous PS mats, PIPAAm-grafted fiber was thicker than ungrafted one. Also, the topography of PS dish surface was changed by the graft of PIPAAm. According to these results, we confirmed that PIPAAm was successfully grafted onto electrospun nanofibrous PS mats and PS dish surfaces.

PIPAAm-grafted surfaces exhibited decreasing contact angles by lowering the temperature from 37 to 20°C, while ungrafted PS surfaces had negligible contact angle changes with changing temperature (Table 1). This result indicates that PIPAAm-grafted surfaces, which are hydrophobic at higher temperature, became remarkably more hydrophilic in response to a temperature reduction due to spontaneous hydration of surface grafted PIPAAm. PIPAAm-grafted PS mats and PIPAAm-grafted PS dishes showed contact angle gaps of 23.77° and 13.33° by temperature change from 37 to 20°C. Water contact angle change of more than 10° which occurred by temperature alteration was enough for cell detachment.

### 3.3. Cell Proliferation on Thermoresponsive Matrices

**Figure 6:**
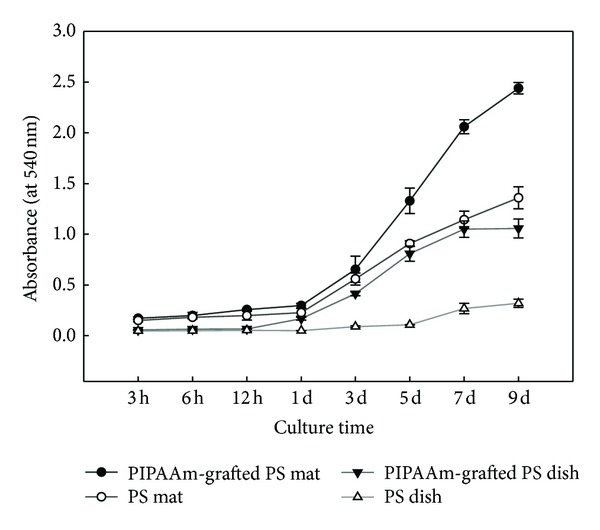
MTT assay of cultured fibroblasts on polystyrene mats and polystyrene dishes.

To demonstrate biocompatibility and cell proliferation, fibroblasts were cultured on PIPAAm-grafted PS surfaces and ungrafted PS surfaces. Attached and spread fibroblasts on nanofibrous PS mat were proliferated more rapidly than those of flat PS dish surface (Figure 6). After 3 hours of culture, initial cell attachment on electrospun nanofibrous PS mat surfaces was higher than PS dish surfaces. Electrospun mat has a higher specific surface area; the three-dimensional structure of electrospun mats gives good metabolism to cells and the surface morphology was rougher than surface of PS dish. For these reasons, cells were attached easily onto electrospun PS mat surfaces. Proliferation of cells on PIPAAm-grafted surfaces was higher than that of ungrafted surfaces. The surface property changed to be hydrophilic by the graft of PIPAAm, which increased compatibility of surfaces to the cells.

### 3.4. Recovery of Cultured Cells

Detachment of single cells from PIPAAm-grafted PS surfaces was induced by low temperature treatment after incubation at 37°C. Almost all of the seeded cells were attached and spread on these surfaces after 5 hours of culture at 37°C. When the culture temperature was reduced to 20°C after 5 h incubation at 37°C, the spread cells became rounded and detached from both PIPAAm-grafted PS dish and nanofibrous mat surfaces. This is because PIPAAm is hydrated below its LCST, producing an expanded, swollen, and hydrophilic surface. This surface property changes weakened cellular adhesion, resulting in spontaneous cell detachment.

**Figure 7:**
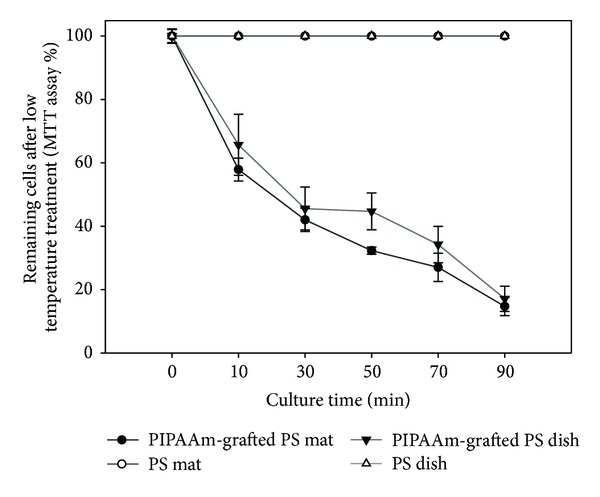
The percentage of remaining cells on ungrafted and PIPAAm-grafted polystyrene surfaces as a function of incubation time at 20°C.

**Figure 8:**
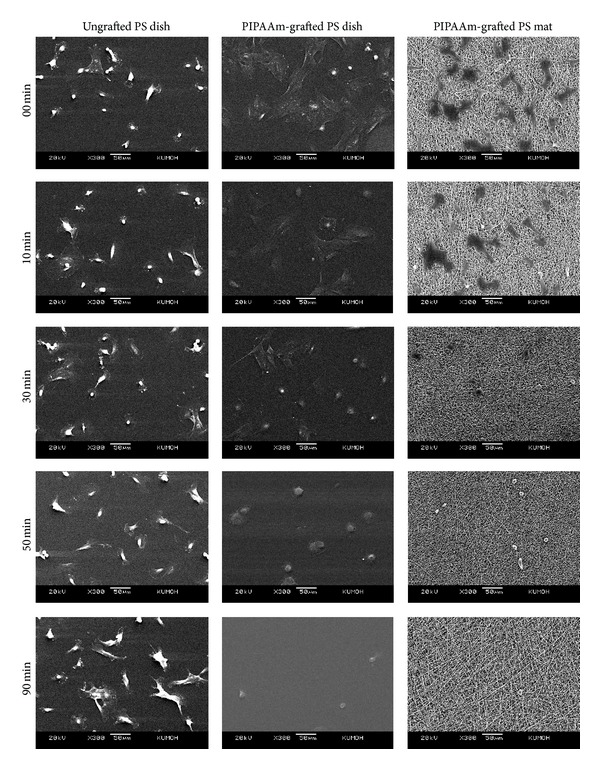
SEM micrographs of fibroblasts detachment from the ungrafted polystyrene dish, PIPAAm-grafted polystyrene dish, and PIPAAm-grafted polystyrene mat surface as a function of incubation time at 20°C.

The percentage of still attached single cells after low temperature treatment decreased rapidly on PIPAAm-grafted surfaces, while there are no cells detached from ungrafted surfaces because of no surface property alternation by reducing culture temperature (Figure 7). Time-lapse images (in minutes) of cell morphology assist the result of MTT assay (Figure 8). Spread cells were more rapidly detached on PIPAAm-grafted PS mat than PIPAAm-grafted PS dish. This difference is probably because of porous structure, the water molecules rapidly reach to grafted PIPAAm from underneath and peripheral to the attached cells, resulting in rapid hydration of grafted PIPAAm molecules and accelerating detachment of the cells. Initial cell attachment, rapid proliferation, and rapid intact cell recovery are important to maintain biological functions and viability of cell source for the fields of tissue engineering and regenerative medicine.

## 4. Conclusions

In this study, PS nanofibrous mats prepared by electrospinning method and subsequent grafting of PIPAAm by electron beam irradiation enabled a rapid cultured cells detachment for biological function and viability of cultured cells. Temperature- responsive surface was developed by introducing thermosensitive PIPAAm chains onto PS nanofibrous mats via electron beam irradiation. Heat treatment was employed to provide bonding points between fibers resulting in increase in mechanical property for sufficient washing process.

Also we expected porous substrate would assist more rapid hydration for functionality of cultured cells. From the noticed results, cells were well attached and proliferated on nanofibrous PS mat more than flat PS dish surface. Also cells were detached more rapidly on PIPAAm-grafted PS nanofiber surfaces than PIPAAm-grafted PS dish surfaces probably due to the effective water supply via existing pores on nanofibers. To maintain biological functions and viability of recovered cells, development of rapid cell recovery system is prerequisite. From this view point, PIPAAm-grafted nanofibrous mats could be a promising tool to recover intact cultured cells.

## Figures and Tables

**Table 1:** Water contact angle (°) of each surface measured by sessile drop method (*n* = 6).

	20°C	37°C
PIPAAm-grafted polystyrene mat	47.83 ± 2.79	71.60 ± 1.82
Ungrafted polystyrene mat	75.83 ± 1.80	74.80 ± 0.45
PIPAAm-grafted polystyrene dish	46.00 ± 1.87	58.33 ± 2.50
Ungrafted polystyrene dish	82.50 ± 1.38	82.80 ± 0.84
